# Adult granulosa cell tumors of bilateral ovaries with pure cystic presentation: A case report and review of literature

**DOI:** 10.1097/MD.0000000000022511

**Published:** 2020-10-02

**Authors:** Bo Wang, Xin Xu, Zhenya Zhao, Dongying Yao, Lei Qi, Yang Zhou

**Affiliations:** Department of Pathology, Xingtai People's Hospital, Hebei Medical University Affiliated Hospital, Xingtai, Hebei, P.R. China.

**Keywords:** cystic granulosa cell tumor, granulosa cell tumor, ovarian cysts, ovarian malignancies, ovary

## Abstract

**Rationale:**

Granulosa cell tumors (GCTs) are rare, hormonally active sex cord-stromal tumors that generally present as solid unilateral ovarian lesions. It's quite uncommon that they present as pure bilateral ovarian cysts. Histopathology remains the gold standard for making a diagnosis of GCTs. However, as the differential diagnosis is difficult, cystic GCTs are frequently misdiagnosed as benign or other cystic tumors either prior to surgery or during pathologic diagnosis. Accordingly, herein, we describe a fairly rare case of bilateral ovarian cystic GCTs, along with a review of the related literature.

**Patient concerns:**

A 43-year-old woman presented with abdominal distension and chronic pain since 1 day. The patient had a history of dysmenorrhea.

**Diagnoses:**

Physical examination revealed palpable bilateral adnexal tumors; ultrasonography revealed cystic and septate masses with a maximum diameter of 7.8 and 10.7 cm, respectively, in the bilateral ovaries. Hormonal analysis revealed that the blood estradiol levels were elevated. Postoperative pathological and immunohistochemical examinations of the surgical specimens revealed a final diagnosis of cystic adult GCTs of the ovaries.

**Interventions:**

The patient first underwent laparoscopic bilateral ovarian cystectomy. On the basis of the final pathological diagnosis report, abdominal total hysterectomy, bilateral oophoro-salpingectomy, and partial omentectomy were then performed. Microscopic examination revealed that there were no residual CGT cells. The patient's federation international of gynecology and obstetrics (FIGO) Stage was IB period.

**Outcomes:**

The surgeries were successful. The tumor was a FIGO Stage IB tumor, and the patient did not require any additional treatment. The patient had been followed-up regularly for 2 years after surgery; she did not experience any complications and remained disease-free.

**Lessons subsections:**

Cystic GCTs should be considered in the differential diagnosis if a female patient shows bilateral ovarian cysts. They are extremely rare ovarian malignant tumors that must be differentiated from other ovarian tumors, especially purely cystic tumors and benign cysts. Although pathological and immunohistochemical findings are important for making the diagnosis, the varying histopathological features on microscope make diagnosis difficult, including tumor cells with luteinization or free cell clusters. The current case highlights the importance of physicians being aware of and suspecting cystic CGTs in similar cases, along with knowing the characteristics of GCTs for the diagnosis and differential diagnosis.

## 1 Introduction

Ovarian granulosa cell tumors (GCTs) are rare, hormonally active sex cord-stromal tumors, accounting for 1% to 5% of all ovarian tumors.^[1]^ They are divided into 2 subtypes: adult granulosa cell tumors (AGCT) and juvenile granulosa cell tumors (JGCT). AGCTs are the more common type, accounting for approximately 95% of all cases; they mainly present in peri- or postmenopausal women aged ≥40 years.^[2]^ JGCTs are the rarer type, accounting for <5% of cases, and they mainly presents in prepubescent girls and women aged <30 years.[^3^,^4^,^5^] On histological examination, the presence of Call-Exner bodies and “coffee-bean” nuclei with a low mitotic activity are typical characteristics of AGCTs. In contrast JGCTs have only a few typical characteristics; immature and atypical nuclei with increased mitotic rates are occasionally observed in JGCTs.^[3]^ Accordingly, GCTs usually present as predominantly solid lesions or solid and cystic lesions, and rarely as pure cystic tumors. Moreover, ovarian cystic GCTs present with nonspecific symptoms that also pose a diagnostic challenge to clinicians, and they often present with appearances similar to other ovarian cystic neoplasms, thereby causing misdiagnosis prior to surgery. Accordingly, histopathology remains the gold standard for diagnosing ovarian cystic GCTs. However, the varying histopathological features on microscope make diagnosis difficult, including tumor cells with luteinization or free cell clusters. As the differential diagnosis is difficult, or pathologists are not familiar with the histologic pattern of their variations, cystic GCTs are misdiagnosed as benign or other cystic tumors either prior to surgery or during pathologic diagnosis. To improve pathologists’ and clinicians’ knowledge regarding the diagnosis and differential diagnosis of cystic GCTs, so as to improve the quality of management of this affection, we describe an unusual case of bilateral ovarian cystic GCTs, along with a review of the related literatures. The current case also further shows how an already rare tumor can present itself in a way which can lead to difficulties determining the final diagnosis and may affect subsequent treatment.

## 2 Case report

A 43-year-old woman who presented with abdominal distension and chronic pain since 1 day showed multilocular pure cystic masses in both the ovaries on radiologic examinations. She had a history of dysmenorrhea, and had no a history of dysfunctional uterine bleeding or menstrual irregularities. This case was treated in accordance with the ethical standards of Xingtai People's Hospital. Written consent was given by the patient for his information to be stored in the hospital database and used for research.

**Figure 1:**
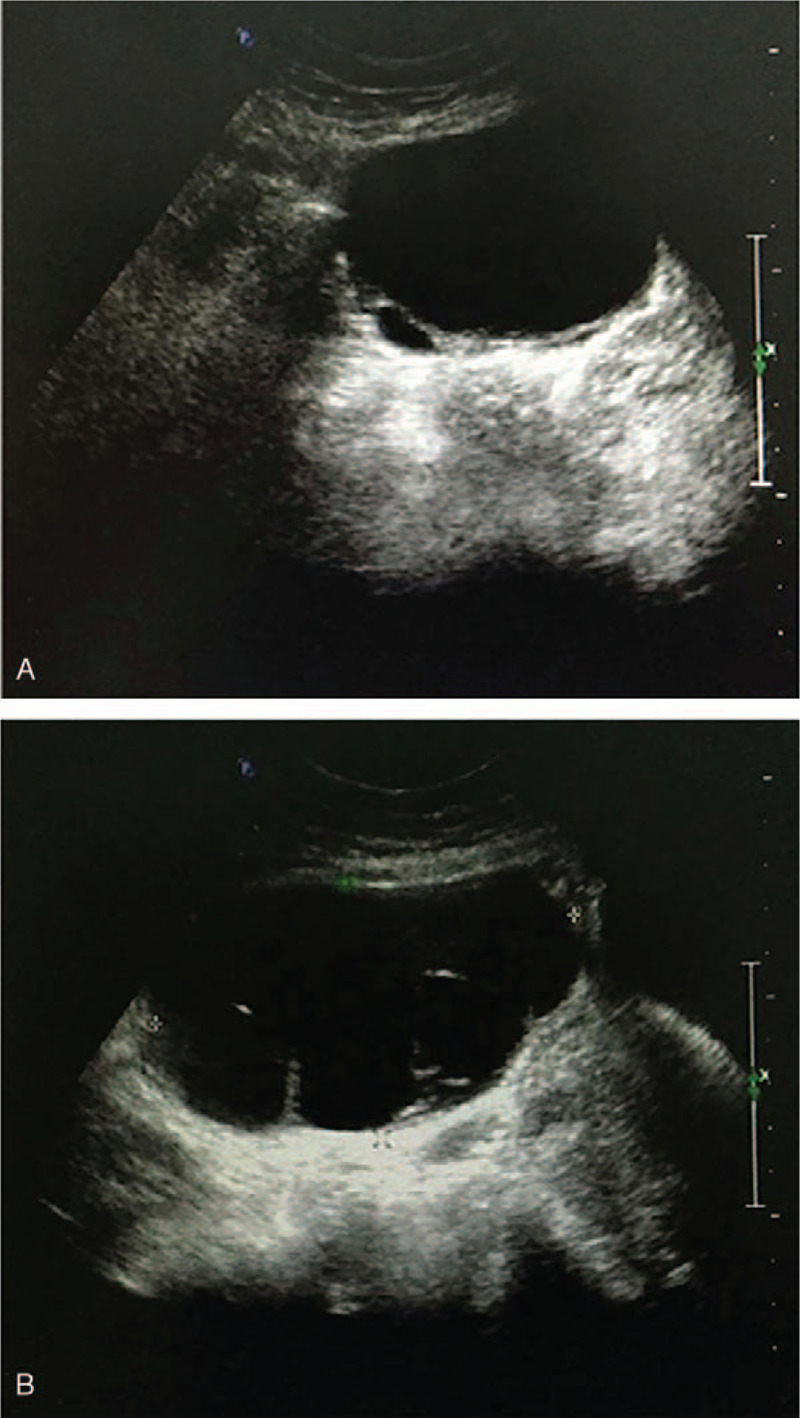
Ultrasonography revealed the completely anechoeic lesions of both ovaries suggestive of a purely cystic consistency. Please note that there were septum in the lesions. (A) Cystic lesion in the left ovary; (B) cystic lesion in the right ovary.

Physical examination revealed a palpable 8 cm, left adnexal tumor and a palpable 10 cm, right adnexal tumor. Ultrasonography revealed a 7.8 cm, cystic septate lesion in the left ovary and a 10.7 cm, cystic, septate lesion in the right ovary; no soft tissues were noted within both the lesions (Fig. 1A and B). Therefore, an initial diagnosis of benign cysts in the bilateral ovaries was made. Hormonal analysis showed that the estradiol levels were elevated to 582 pg/mL (reference range 27–433 pg/mL). The levels of other tumor markers, including alpha-fetoprotein (AFP), CA199, CA125, and carcinoembryonic antigen (CEA), were not elevated. Chest radiography did not reveal any remarkable findings. The patient underwent laparoscopic bilateral ovarian cystectomy, and the cysts were not ruptured during cystectomy. The findings on frozen section analysis of the ovarian tumors indicated that GCTs could be a possibility. Accordingly, the patient's kin decided to choose the next surgical procedures after obtaining a final pathological diagnosis.

**Figure 2:**
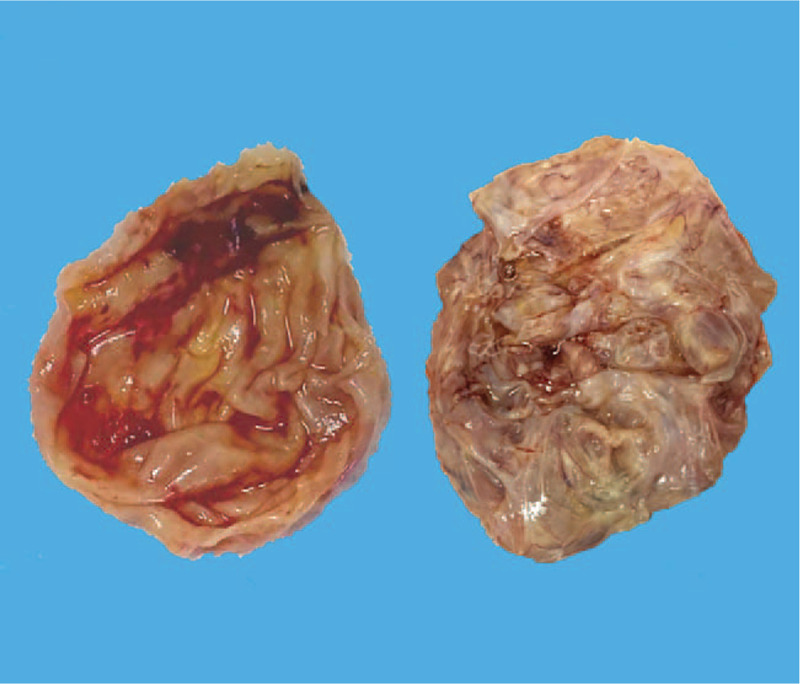
Gross pathological specimen showing cut surface of the ovarian cyst wall. The cyst wall was irregular and thick.

**Figure 3:**
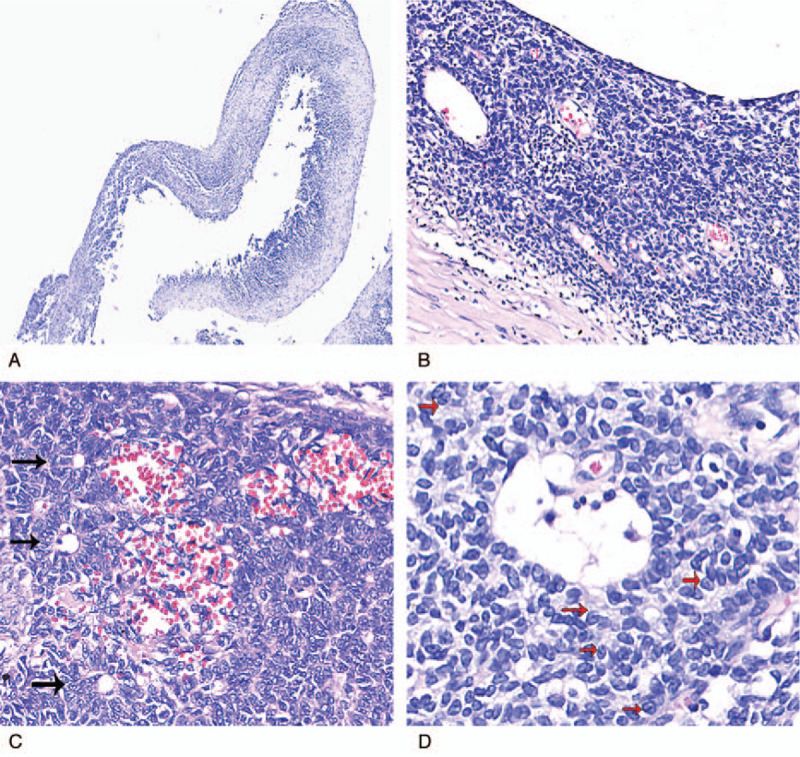
Photomicrograph, hematoxylin and eosin staining. (A) The cyst was lined by a simple layer to multilayer epithelium cells resting on the thick fibrous wall. Magnification, ×40. (B) The multilayer lining epithelium cells of the cyst wall; they surrounded the macrofolliculars with eosinophilic material. Magnification, ×200. (C) Call-Exner bodies were noted. Magnification, ×400. (D) Cuboidal to columnar epithelium cells resting on the cyst wall; the epithelium cells had fine chromatin and round to oval nuclei with single small nucleoli. Some nuclei show longitudinal grooves. Magnification, ×400. (E) At some cystic regions, many epithelium cells with luteinization, which had abundant, eosinophilic or clear cytoplasm, and round nuclei that without nuclear grooves. Magnification, ×400.

**Figure 4:**
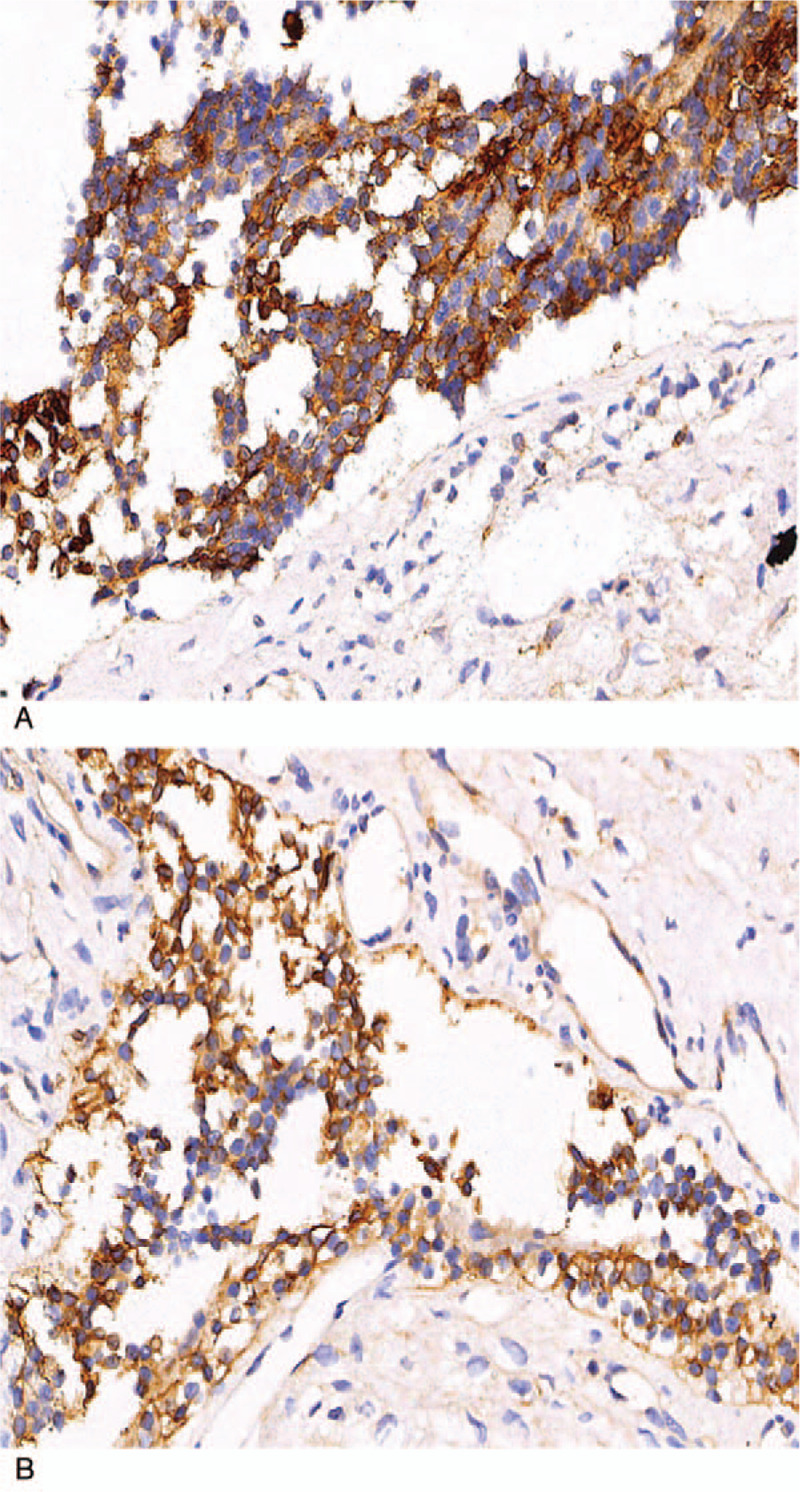
Photomicrograph, immunohistochemical staining showing the epithelial cells were positive for (A) alpha-inhibi. Magnification, ×400. (B) Vimentin. Magnification, ×400.

The gross appearance of the bilateral ovarian tumors showed multiloculated cysts (Fig. 1) accompanied by intracystic yellowish, clear liquid. The cyst wall was irregular and thick (Fig. 2). Microscopic examination of the cyst wall revealed that the cyst wall was lined by cuboidal to columnar epithelium cells resting on the thick fibrous wall (Fig. 3A and B). Call-Exner bodies were also noted (Fig. 3C). The epithelial cells had fine chromatin and round to oval nuclei with single small nucleoli. Some nuclei, known as “coffee-bean” nuclei, showed pale, round longitudinal grooves (Fig. 3D). We also observed many epithelial cells with luteinization, which had abundant, eosinophilic, or clear cytoplasm, and round nuclei without nuclear grooves (Fig. 3E). Mitotic figures were rare (<2 mitoses per 10 high-power fields). The epithelial cells detached or separated from the cyst wall, leading to cyst walls without epithelium as well as many free cell clusters at some cystic regions. On immunohistochemical analysis, the epithelial cells were positive for alpha-inhibin (Fig. 4A), vimentin (Fig. 4B), CD99 and pan-cytokeratin (AE1/AE3), and patchily stained for P53; the proliferating cell nuclear antigen Ki-67 index was approximately 10%. The epithelial cells were negative for CEA, epithelial membrane antigen (EMA), smooth muscle actin (SMA), S100, synaptophysin (Syn), and chromogranin A (CgA). Thus, the findings supported a final diagnosis of cystic AGCTs in the bilateral ovaries.

We performed abdominal total hysterectomy, bilateral oophoro-salpingectomy, and partial omentectomy. Microscopic examination showed that there were no residual GCT cells. In addition, as the tumor was a FIGO Stage IB tumor no additional treatment as required. The patient has been followed-up regularly and disease-free for 2 years after surgery.

## 3 Discussion

GCTs are rare sex cord-stromal tumors with a low malignant potential. They present as predominantly unilateral solid masses with varying amounts of hemorrhage and necrosis, which is responsible for causing the cystic components. However, unilateral pure cystic tumors are rare.[^6^,^7^,^8^] And herein, we report an even rarer tumor type: bilateral cystic GCTs of the ovaries.

GCTs are hormonally active tumors, and approximately 75% of them secrete estrogen, which is responsible for endometrial hyperplasia, endometrial carcinoma, and abnormal uterine bleeding.^[8]^ The hyperestrogenic state in the JGCT variety is responsible for precocious puberty.[^9^,^10^] Therefore, abdominal distension, pain, and the presence of a mass are the most common symptoms of GCTs. In addition, few cases of GCTs that secrete androgen are responsible for features of virilization, including atrophy of the vulva and hirsutism.[^8^,^9^] In contrast, a few clinical cases of GCTs do not secrete any hormones, so the patients do not show any symptoms of endocrine disorders.^[11]^ In our this case, the patient presented with abdominal distension, chronic pain, and abdominal masses; hormonal analysis showed that the estradiol levels were elevated. These symptoms were consistent with the reported in the literature.

The radiological criteria indicative of malignant ovarian masses include irregular, thick walls and septa; papillary projections; and solid, echogenic foci.^[12]^ The current case showed irregular, thick walls, and septa. These radiological characteristics indicate that malignant tumor types should be considered in the differential diagnosis of purely cystic ovarian lesions.

Cystic GCTs are subdivided into AGCT and JGCT, indicating not only the typical age of the patient and the differentiating histologic features, but also the differing natural history. However, both forms of GCTs may occur in any age group. On reviewing the literature, we found that two-thirds of all AGCT cases present in postmenopausal women, with a peak incidence between 50 and 55 years,^[10]^ while 1% of AGCT cases were observed in prepubescent girls.^[13]^ JGCTs mainly present in prepubescent girls and women aged <30 years, with more than a half of the cases in patients aged <10 years.^[13]^ The classic features of AGCT on histological examination include Call-Exner bodies and “Coffee-bean” nuclei along with a low mitotic rate. Call-Exner bodies are gland-like structures that appear similar to ovarian follicles. “Coffee-bean” nuclei are pale, round, and longitudinally grooved. In contrast, JGCTs have only a few typical findings. JGCTs have fewer Call-Exner bodies, and the gland-like structures resembling ovarian follicles are irregular in size and shape. In addition, JGCTs have immature nuclei with atypia and increased mitotic activity. On immunohistochemistry, granulosa cells display stable expression of alpha-inhibin, vimentin, and CD99. Based on the alpha-inhibin, vimentin and CD99 positive together, pathologists always can be confident in the diagnosis of GCT. In the current case, the epithelial cells of the ovarian tumors were also positive for alpha-inhibin, vimentin, and CD99.

The differential diagnosis for GCTs should include other cystic masses and malignant tumors of the ovary (e.g., follicular cysts, cystadenoma, carcinoid, adenocarcinoma, and ovarian thecoma). We have discussed the characteristics of these tumors below.

Cystic GCTs include unilocular type and luteinized type that are easily misdiagnosed as follicular cysts and luteinized follicular cysts.[^14^,^15^] As the cyst wall of some GCTs is lined by simple or a few stratified solid tumor cell nests, misdiagnosis is a possibility. On histological examination, the cyst walls of follicular cysts are lined by 2 types of cells: simple or stratified granular cells (approximately 4 layers) in the inner layer and thicker theca cells in the outer layer. The granular layer might be completely absent in some regions. Moreover, follicular cysts without Call-Exner bodies and atypical cells have been observed.

Cystadenomas might be misdiagnosed. The cyst wall of cystadenomas is lined by mucous or/and serous epithelium. A cystadenoma comprising follicular cysts has been reported; this type of cystadenomas have increased the difficulty diagnosis.^[16]^

Carcinoids are often misdiagnosed because both the small blue tumor cells and the cell arrangement modes are similar to GCTs.^[17]^ However, carcinoid cells are small and round, with a monotonous appearance; they do not have “coffee-bean” nuclei. On immunohistochemical examination, carcinoids are positive for neuroendocrine markers such as Syn, CgA, and CD56.

As Call-Exner bodies are gland-like structures, it is important to differentiate between cystic GCTs and adenocarcinomas. Adenocarcinoma shows the presence of cellular atypia and salient mitotic figures, and it does not show “coffee-bean” nuclei. On ommunohistochemical examination, adenocarcinoma cells are positive for epithelial membrane antigen and cytokeratin.^[18]^

Unilocular cystic GCTs are easily misdiagnosed as ovarian thecoma because the granular cells have round nuclei without nuclear grooves and abundant eosinophilic or clear cytoplasm. Elastic fiber staining can be used for the differential diagnosis. Elastic fiber staining showed the presence of elastic fibers around nests of granular cells, while elastic fibers were observed around every thecoma cell.

Furthermore, an ovarian multiloculated cyst comprising GCT, mature cystic teratoma, and serous cystadenoma has been reported.^[16]^ Accordingly, pathologists should evaluate a sufficient number of sections before making a diagnosis, thus avoid missed diagnosis, misdiagnosis, and mistreatment.

To date, the standard treatment for GCTs remains resection of the tumor; nevertheless, several other treatments have been performed, as needed, including chemotherapy, radiotherapy, gonadotrophin-releasing hormone agonists, and aromatase inhibitors. GCTs have the clinical features of low malignancy potential, local invasion, and/or recurrence. The prognostic factors predicting recurrence include advanced clinical stages, larger tumor size, nuclear atypia, and higher mitotic rates on pathologic examination. GCTs can recur many years after diagnosis, and the longest reported time from primary presentation to recurrence was 37 years.^[19]^ Unlike AGCTs, JGCTs are relatively benign with excellent survival rates that can be as high as 97%^[13]^; moreover, the rates of recurrence are higher for AGCTs.^[20]^ Because of the differences in the prognosis, accurate diagnosis is necessary for patients; in addition, clinical long-term follow-up is required, as GCTs can recur.

In conclusion, cystic GCTs are rare ovarian malignancies, and a few cases can present as pure cystic tumors and may occur in the bilateral ovaries. Till date, there have been only a few reports of cystic GCTs on the bilateral ovaries. Careful histopathological examination remains the gold standard for diagnosis. As the clinical characteristics of GCTs are unknown and accurate diagnosis is difficult, the description of cystic GCTs reported herein-along with the literature review of the features of cystic GCTs from published case reports—add to pathologists’ and clinicians’ knowledge regarding the diagnosis and differential diagnosis of cystic GCTs, thus to improve the quality of management of this affection. Moreover, the current case further shows how an already rare tumor can present itself in a way which can lead to difficulties determining the final diagnosis and may affect subsequent treatment. In addition, on the basis of the findings of the current case, we show that cystic GCTs have thick, irregular walls, and septa. Hence, bilateral, purely cystic, ovarian lesions should be included in the differential diagnosis of malignant ovarian lesions. We believe this paper will be helpful for diagnosing future cases of GCTs.

## Acknowledgments

The authors would like to thank Editage (www.editage.com) for English language editing.

## Author contributions

Bo Wang is the first author of this report, she also analyzed the data and wrote the manuscript. Xin Xu took the role of pathological diagnosis. Zhenya Zhao, Dongying Yao, and Lei Qi assisted with literature collection and diagnosis, Yang Zhou provided imaging description and figures. All authors have read and approved the final manuscript.
